# Gill developmental program in the teleost mandibular arch

**DOI:** 10.7554/eLife.78170

**Published:** 2022-06-28

**Authors:** Mathi Thiruppathy, Peter Fabian, J Andrew Gillis, J Gage Crump

**Affiliations:** 1 Eli and Edythe Broad California Institute for Regenerative Medicine Center for Regenerative Medicine and Stem Cell Research, Department of Stem Cell Biology and Regenerative Medicine, University of Southern California Keck School of Medicine Los Angeles United States; 2 Marine Biological Laboratory Woods Hole United States; 3 Department of Zoology, University of Cambridge Cambridge United Kingdom; Northwestern University United States; California Institute of Technology United States

**Keywords:** Pseudobranch, Gill, jaw, mandibular arch, cranial neural crest, Zebrafish

## Abstract

Whereas no known living vertebrate possesses gills derived from the jaw-forming mandibular arch, it has been proposed that the jaw arose through modifications of an ancestral mandibular gill. Here, we show that the zebrafish pseudobranch, which regulates blood pressure in the eye, develops from mandibular arch mesenchyme and first pouch epithelia and shares gene expression, enhancer utilization, and developmental *gata3* dependence with the gills. Combined with work in chondrichthyans, our findings in a teleost fish point to the presence of a mandibular pseudobranch with serial homology to gills in the last common ancestor of jawed vertebrates, consistent with a gill origin of vertebrate jaws.

## Introduction

Gills are the major sites of respiration in fishes. They are composed of a highly branched system of primary and secondary filaments, housing blood vessels, a distinct type of cellular filament cartilage, pillar cells (specialized endothelial cells), and epithelial cells maintaining ionic balance. In teleost gills, two rows of filaments are anchored to a prominent gill bar skeleton. Both the filaments and supportive gill bars develop from the embryonic pharyngeal arches that consist of mesenchyme of neural crest and mesoderm origin and epithelia of endodermal and ectodermal origin (Fabian et al., 2022; Mongera et al., 2013). The third and more posterior arches generate gills in most fishes. The second (hyoid) arch also forms a hemibranch (one row of gill filaments) in the jawless lamprey fish (Dohrn, 1882; Gaskell, 1908), in cartilaginous and various non-teleost fishes (e.g. coelacanth, lungfishes, sturgeon, and gar), but not in teleost fishes (Goodrich, 1930; Jollie, 1962). A classical theory for the origin of jaws posits that an ancestral gill support skeleton in the mandibular arch was repurposed for jaw function (Gegenbaur et al., 1878). However, extant agnathans (the cyclostomes lamprey and hagfish) lack a mandibular gill (Cole, 1905; Mallatt, 1996), and fossil evidence for ancestral vertebrates with a mandibular gill is scant. Whereas exceptional soft tissue preservation of *Metaspriggina walcotti* from the Cambrian Burgess Shale had suggested a dorsoventrally segmented cartilaginous gill bar in the presumptive mandibular arch, gill filaments were not observed (Morris and Caron, 2014).

The pseudobranch is an epithelial structure located just behind the eye that has been proposed to regulate ocular blood pressure and/or have an endocrine function (Jollie, 1962). While it shares an anatomical resemblance to gill filaments and is found in many jawed fishes (Dohrn, 1882), its embryonic arch origins remain debated (Miyashita, 2016). In parallel work in little skate, we identify a mandibular arch origin of the pseudobranch in chondrichthyans (Hirschberger and Gillis, 2022), which form one branch of jawed vertebrates. Whether the mandibular origin of the pseudobranch is conserved across vertebrates, including bony fishes, remained unknown. Another important question is whether the pseudobranch and gills can be considered serially homologous, i.e., representing morphologically related structures that arise through shared developmental and genetic mechanisms. Through lineage tracing and genetic analyses in zebrafish here, and lineage analysis in skate (Hirschberger and Gillis, 2022), we infer that the pseudobranch is a mandibular arch-derived serial homolog of the gills that was present in at least the last common ancestor of jawed vertebrates.

## Results

**Figure 1.**
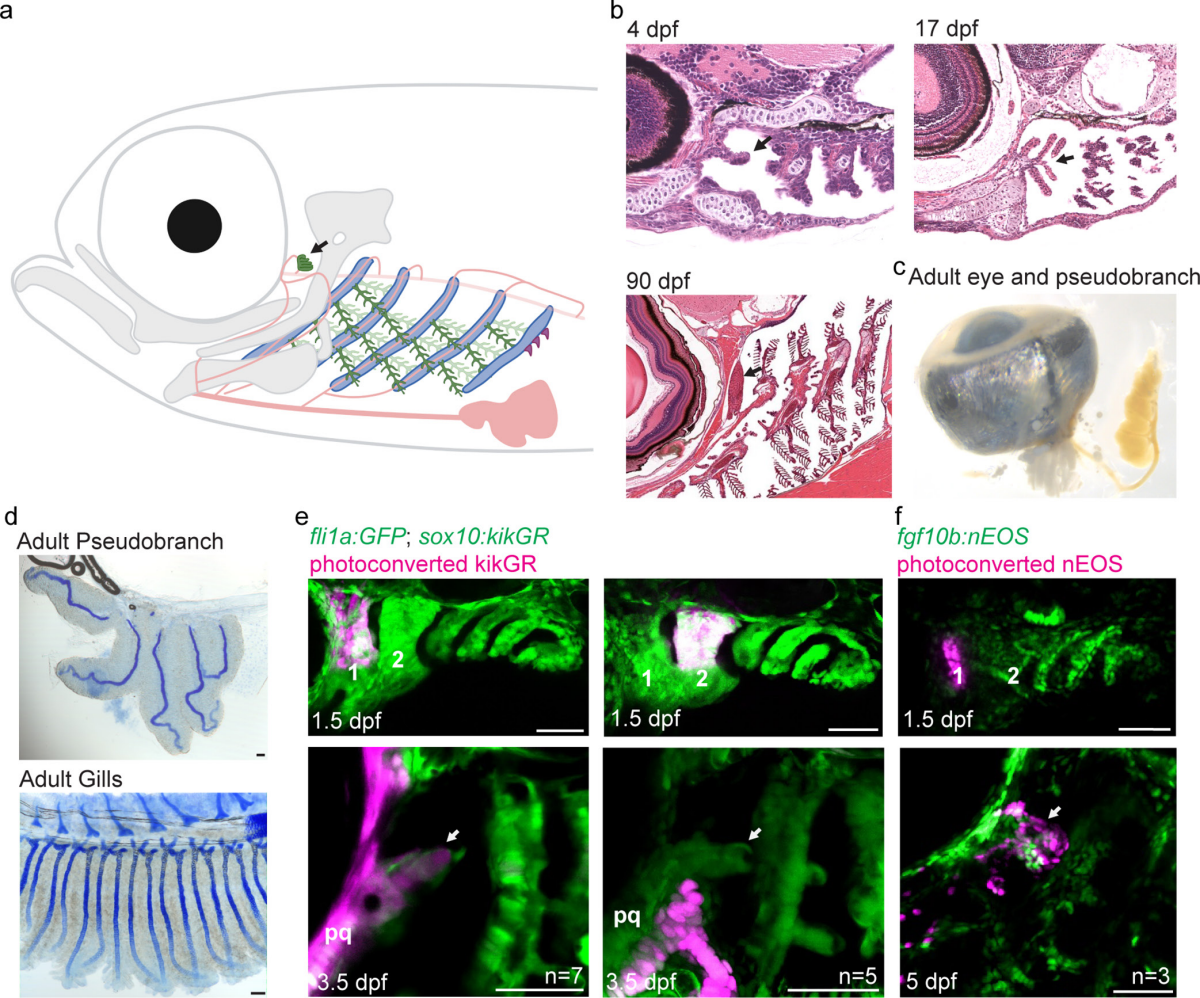
The zebrafish pseudobranch derives from mandibular arch mesenchyme and first pouch epithelia. (**a**), Schematic showing the pseudobranch (arrows), gill filaments (branched green structures) connected to gill bars (blue), teeth (purple), vasculature (pink), and jaw and jaw-support skeleton (gray). (**b**) Hematoxylin and Eosin-stained sections show emergence of the pseudobranch bud at 4 dpf (adapted from https://bio-atlas.psu.edu/zf/view.php?atlas=5&s=41), five filaments at 17 dpf (adapted from https://bio-atlas.psu.edu/zf/view.php?atlas=65&s=1738), and the fused pseudobranch at 90 dpf (adapted from https://bio-atlas.psu.edu/zf/view.php?atlas=29&s=312). (**c**) Dissected adult pseudobranch shows the ophthalmic artery connecting it to the eye. (**d**) Alcian staining shows five cartilage rods in the pseudobranch and similar cartilage in gill primary filaments. (**e**) Photoconverted kikGR-expressing mesenchyme (red) from the dorsal first arch (numbered) at 1.5 dpf contributes to the palatoquadrate cartilage (pq) and pseudobranch mesenchyme (arrow) at 3.5 dpf. Photoconverted dorsal second arch cells do not contribute to the pseudobranch. In green, *fli1a*:*GFP* labels the vasculature and neural crest-derived mesenchyme, with mesenchyme also labeled by unconverted *sox10:kikGR*. (**f**) In *fgf10:nEOS* embryos, photoconversion of first pouch endoderm (numbered) at 1.5 dpf labels the pseudobranch epithelium (arrow) at 5 dpf. n numbers denote experimental replicates in which similar contributions were observed. Scale bars, 50 µm.

In zebrafish, the pseudobranch is located anterior to the gill filaments and connected to the eye via the ophthalmic artery (Figure 1a, c), as described for other fishes (Laurent and Dunel-Erb, 1984). The pseudobranch appears in histological sections as a small bud behind the eye at 4 days post-fertilization (dpf) (Figure 1b). Examination of *Sox10:Cre; acta2:loxP-BFP-Stop-loxP-dsRed* zebrafish shows this bud to be composed of a core of Cre-converted dsRed+ neural crest-derived cells ensheathed by unconverted BFP+ epithelia (Figure 1—figure supplement 1a). The position of this bud corresponds to *kdrl:mCherry* labeling of a branch of the first aortic arch that likely gives rise to the ophthalmic artery (Figure 1—figure supplement 1b). At 17 dpf, the pseudobranch is composed of five distinct filaments that resemble the primary gill filaments, with the five filaments merging to form a single pseudobranch by adult stages (90 dpf) (Figure 1b). Alcian Blue staining reveals that the adult pseudobranch contains five cartilage rods, reflecting the five fused filaments, with this cartilage resembling the specialized filament cartilage seen in the gills (Figure 1d; Fabian et al., 2022).

**Figure 1—figure supplement 1.**
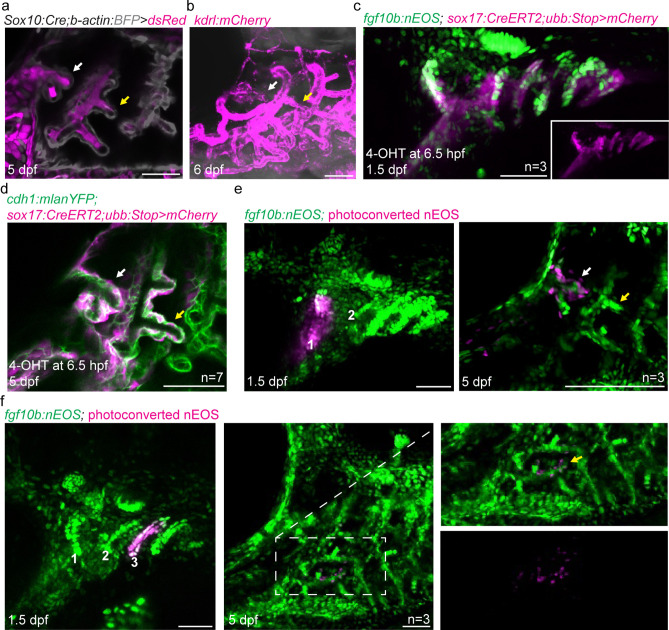
Development of zebrafish pseudobranch and lineage analysis of gill filament epithelia. (**a**) In *Sox10:Cre; acta2:loxP-BFP-Stop-loxP-dsRed* fish at 5 dpf, the developing pseudobranch (white arrow) and gill buds (yellow arrow) consist of Cre-converted dsRed+ neural crest-derived mesenchyme (magenta) and unconverted BFP+ epithelia (gray). (**b**) At 6 dpf, *kdrl:mCherry* labeling of vasculature reveals a branch of the first aortic arch in the position of the pseudobranch (white arrow), and branches of the posterior aortic arches in the positions of the gills (yellow arrow). (**c**) Endoderm is labeled in red by adding 4OH-tamoxifen to *sox17:CreERT2; ubb:loxP-Stop-loxP-mCherry* fish at 6.5 hr post-fertilization to induce Cre recombination that removes the Stop cassette and allows mCherry expression (mCherry channel alone shown in inset). Co-localization shows *fgf10b:nEOS* expression (green) in endodermal pouches. (**d**), Endoderm labeling by addition of 4OH-tamoxifen to *sox17:CreERT2; ubb:loxP-Stop-loxP-mCherry* fish at 6.5 hr post-fertilization results in contribution to *cdh1:mlanYFP*+ pseudobranch epithelium at 5 dpf. (**e,f**) In *fgf10:nEOS* embryos, photoconversion of first pouch endoderm (and some more ventral mandibular cells) at 1.5 dpf labels pseudobranch (white arrow) but not gill epithelia (yellow arrow) at 5 dpf in (**e**), and conversion of third pouch cells labels the first gill filament epithelium (yellow arrow, boxed region magnified to right and shown in merged and red-only channels) in (**f**). Scale bars, 50 µM.

To determine from which arch the pseudobranch arises, we performed short-term lineage tracing using a photoconvertible *sox10:kikGR* reporter expressed in neural crest-derived mesenchyme. Photoconversion of dorsal first arch mesenchyme at 1.5 dpf labeled the pseudobranch mesenchymal bud at 3.5 dpf, as well as the palatoquadrate cartilage, a known first arch derivative; photoconversion of dorsal second arch mesenchyme did not label the pseudobranch (Figure 1e). To trace the epithelial origins of the pseudobranch, we performed short-term lineage tracing using *fgf10b:nEOS*, in which the photoconvertible nuclear-EOS protein is expressed in endodermal pouch epithelia (Figure 1—figure supplement 1c). Photoconversion of first pouch endoderm at 1.5 dpf labeled pseudobranch epithelia at 5 dpf (Figure 1f; Figure 1—figure supplement 1e), similar to labeling of first gill filament epithelia after photoconversion of third pouch endoderm (Figure 1—figure supplement 1f). We also confirmed endodermal origin of *cdh1:mlanYFP*+ pseudobranch epithelia by 4OH-tamoxifen-mediated conversion of early endoderm in *sox17:CreERT2; ubb:loxP-Stop-loxP-mCherry* zebrafish (Figure 1—figure supplement 1d). The pseudobranch therefore arises from mandibular arch neural crest-derived mesenchyme and first pouch endodermal epithelia.

**Figure 2.**
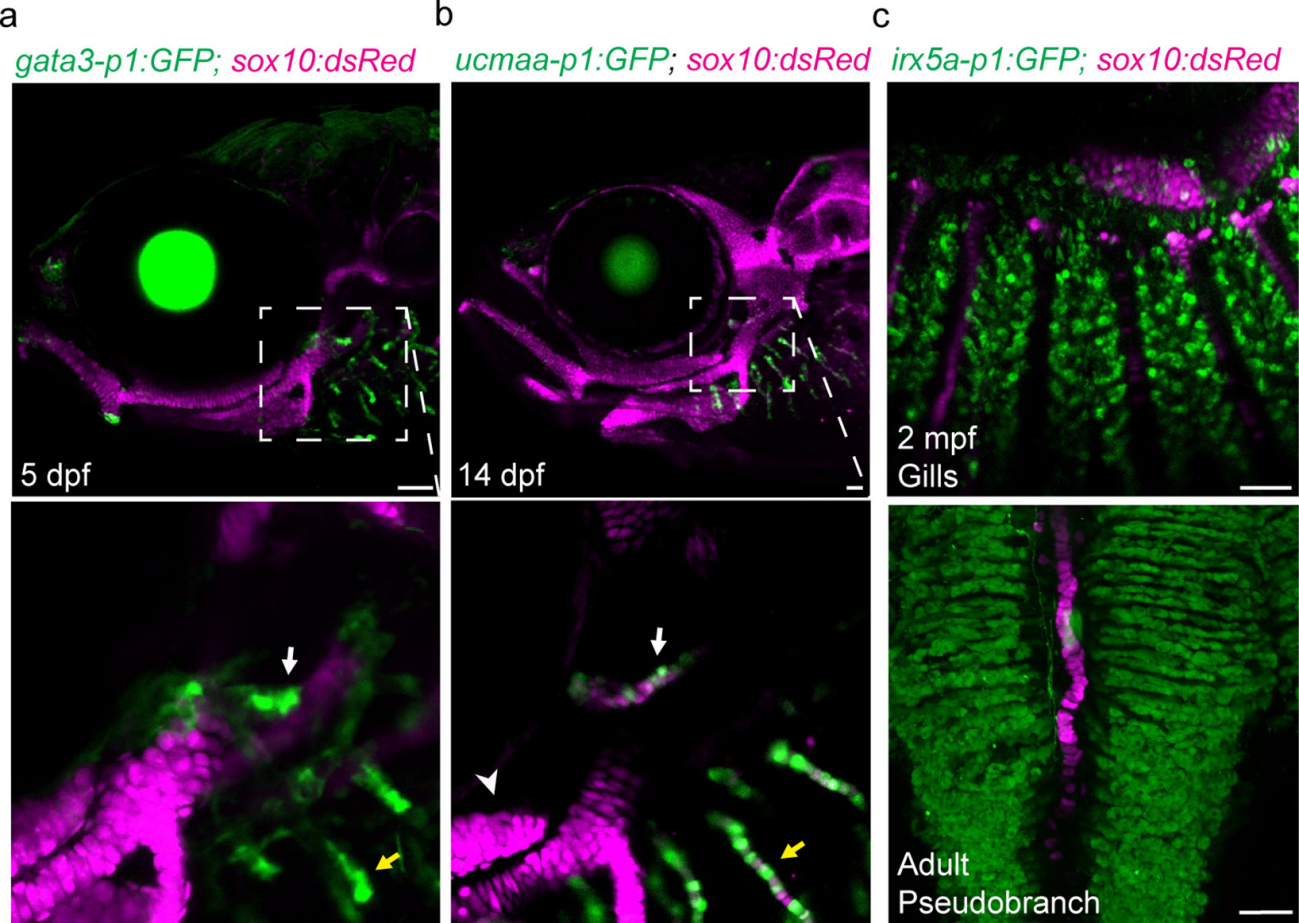
Shared regulatory program for pseudobranch and gill development. (**a-c**) In the pseudobranch (white arrows) and gill filaments (yellow arrows), *gata3-p1:GFP* labels growing buds, *ucmaa-p1:GFP* labels cellular cartilage (distinct from hyaline cartilage, arrowhead), and *irx5a-p1:GFP* labels pillar cells. *sox10:dsRed* labels cartilage for reference. Images in (**b**) and (**c**) are confocal projections, with magnified regions shown below in single sections for *gata3-p1:GFP* and *ucmaa-p1:GFP*. Scale bars, 50 µM.

In skate, the pseudobranch and gills share expression of *foxl2*, *shh*, *gata3*, and *gcm2* (Hirschberger and Gillis, 2022). To test whether this reflects shared gene regulatory mechanisms indicative of serial homology, we examined activity of several gill-specific enhancers (Fabian et al., 2022). At 5 dpf, the gata3-p1 enhancer drives GFP expression in the growing tips of both the pseudobranch and gill buds (Figure 2a). At 14 dpf, the ucmaa-p1 enhancer, active in gill filament but not hyaline cartilage in the face, drives GFP expression in both pseudobranch and gill filament cartilage (Figure 2b), as seen for endogenous expression of *ucmaa* (Figure 3—figure supplement 1a). In our single-cell chromatin accessibility analysis of neural crest-derived cells (Fabian et al., 2022), we also identified an *irx5a* proximal enhancer selectively accessible in pillar cells, a specialized type of endothelial cell in the gill secondary filaments (Figure 3—figure supplement 2a). At 13, 20, and 60 dpf and one-year-old adult fish, the irx5a-p1 enhancer drives GFP expression in pillar cells of the pseudobranch and gills (Figure 2c; Figure 3—figure supplement 2b, c). These findings show that cells with similar gene expression and cis-regulatory architecture are present in both the pseudobranch and gills of zebrafish.

**Figure 3.**
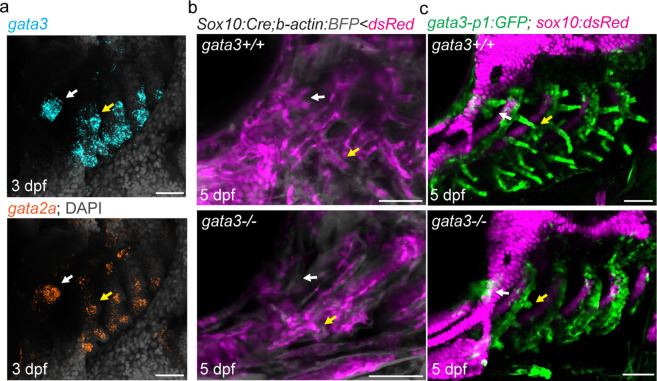
Pseudobranch and gill development requires *gata3* function. (**a**) Similar expression of *gata3* and *gata2a* in developing pseudobranch (white arrows) and gill regions (yellow arrows). (**b**) *Sox10:Cre; acta2:loxP-BFP-Stop-loxP-dsRed* labels Cre-converted dsRed+ neural crest-derived mesenchyme (magenta) and unconverted BFP+ epithelia (gray). (**c**) *gata3-p1:GFP* labels pseudobranch and gill filament buds, and *sox10:dsRed* labels cartilage. For both (**b**) and (**c**), 3/3 *gata3* mutants displayed reduced formation of the pseudobranch (white arrows) and gill filaments (yellow arrows), compared to 3 controls each. Scale bars, 50 µM.

Zebrafish mutant for *gata3* fail to form gill buds (Sheehan-Rooney et al., 2013), and single-cell chromatin accessibility analysis of neural crest-derived cells had implicated *gata3* and *gata2a* in development of gill filament cell type differentiation (Fabian et al., 2022). We find that *gata3* and *gata2a* are prominently expressed in both the developing pseudobranch and gill buds at 3 and 5 dpf (Figure 3a; Figure 3—figure supplement 1b, c). The pseudobranch is also much reduced in *gata3* mutants at 5 dpf, with fewer neural crest-derived cells labeled by *Sox10:Cre; acta2:loxP-BFP-Stop-loxP-dsRed* or *gata3-p1:GFP* contributing to both the pseudobranch and gills (Figure 3b and c). Similar genetic dependency of the pseudobranch and gills further supports serial homology.

**Figure 3—figure supplement 1.**
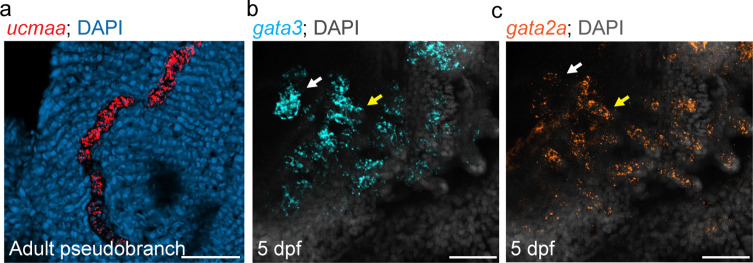
Pseudobranch shares gene expression with gill filaments. (**a**) Expression of *ucmaa* in pseudobranch cartilage from one-year-old fish. (**b,c**) Developing pseudobranch (white arrows) and gill buds (yellow arrows) express *gata3* and *gata2a* at 5 dpf. DAPI labels nuclei in blue (**a**) or white (**b,c**). Scale bars, 50 µM.

**Figure 3—figure supplement 2.**
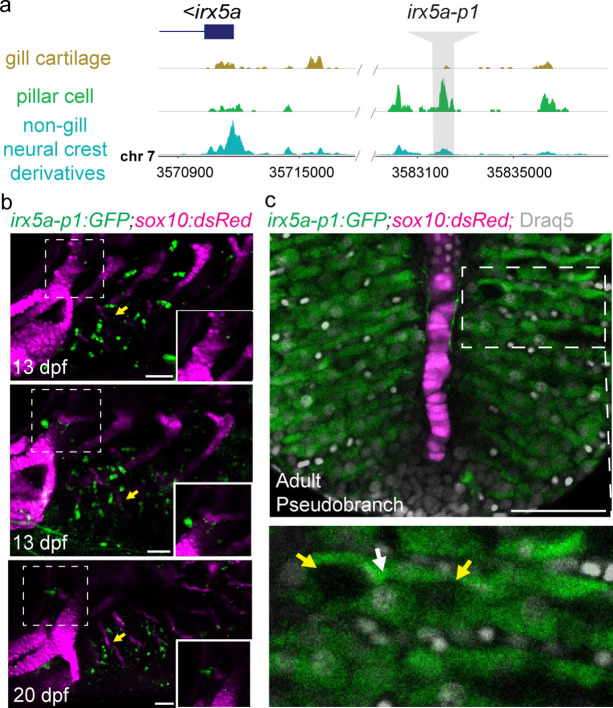
The pseudobranch shares *irx5a-p1* pillar cell enhancer activity with gill filaments. (**a**) An intergenic irx5a-p1 region displays accessible chromatin specifically in pillar cells at 60 dpf. (**b**) irx5a-p1 drives GFP expression in pillar cells of the developing gills (yellow arrows) and pseudobranch (insets). (**c,d**) Dissected pseudobranch from a 60 dpf *irx5a-p1:GFP* adult shows GFP-positive pillar cells adjacent to a core of filament cartilage. The boxed region magnified below depicts an individual pillar cell (white arrow) flanked by characteristic lacunae (yellow arrows). Cartilage is labeled by *sox10:dsRed* in (**b**) and (**c**). Scale bars, 50 µM.

## Discussion

Our findings that both a cartilaginous and teleost fish have a mandibular gill-like pseudobranch suggest that the last common ancestor of jawed vertebrates did so as well, thus providing plausibility to the model that jaws evolved from a gill-bearing mandibular arch. The absence of a pseudobranch in extant agnathans (i.e. lamprey and hagfish) (Cole, 1905; Mallatt, 1996) suggests that either the pseudobranch arose along the gnathostome stem (i.e. prior to the divergence of cartilaginous and bony fishes), or that it was an ancestral feature of vertebrates that has been lost in cyclostomes. The latter would be analogous to loss of the hyoid hemibranch gill during teleost fish evolution (Goodrich, 1930; Jollie, 1962), consistent with our failure to observe gill filament gene expression or transgene activity in the hyoid arch of zebrafish.

Whereas our data clearly point to the filament systems of the pseudobranch and gills being serially homologous, the major skeletal bars supporting the jaws and gills (not to be confused with the gill filament cartilage) appear to develop largely independently from the filaments. Unlike the gill filaments, the zebrafish pseudobranch is not attached to a major skeletal bar. Conversely, the skeletal bars derived from the seventh arch of zebrafish lack gill filaments and instead anchor pharyngeal teeth, and no gill filaments were observed with the fossilized rostral-most gill bar of *M. walcotti* (Morris and Caron, 2014). In addition, *gata3* loss affects the pseudobranch and gill filaments but not the gill bars (Sheehan-Rooney et al., 2013). It is therefore possible that, rather than the pseudobranch evolving from an ancestral mandibular gill whose gill bar was transformed into the jaw skeleton, the pseudobranch arose independently after appearance of the jaw by co-option of a gill filament developmental program. While we demonstrate gill-like developmental potential of the mandibular arch in extant vertebrates, whether an ancestral mandibular gill bar gave rise to vertebrate jaws awaits more definitive fossil evidence.

## Materials and methods

**Key resources table:** 

Reagent type (species) or resource	Designation	Source or reference	Identifiers	Additional information
Gene (*Danio rerio*)	*ucmaa*		Ensembl: ENSDARG00000027799	
Gene (*Danio rerio*)	*gata3*		Ensembl: ENSDARG00000016526	
Gene (*Danio rerio*)	*gata2a*		Ensembl: ENSDARG00000059327	
Gene (*Danio rerio*)	*irx5a*		Ensembl: ENSDARG00000034043	
Genetic reagent (*Danio rerio*)	Tübingen	ZIRC	RRID:ZIRC_ZL57	Wildtype strain of zebrafish
Genetic reagent (*Danio rerio*)	*Tg(fli1a:eGFP)^y1^*	Lawson and Weinstein, 2002		
Genetic reagent (*Danio rerio*)	*Tg(sox10:kikGR)^el2^*	Balczerski et al., 2012		
Genetic reagent (*Danio rerio*)	*Tg(ucmaa_p1:GFP, cryaa:Cerulean)^el851^*	Fabian et al., 2022		
Genetic reagent (*Danio rerio*)	*Tg(gata3_p1:GFP, cryaa:Cerulean)^el858^*	Fabian et al., 2022		
Genetic reagent (*Danio rerio*)	*Tg(fgf10b:nEOS)^el865^*	Fabian et al., 2022		
Genetic reagent (*Danio rerio*)	*Tg(–3.5ubb:loxP-STOP-loxP-mCherry)^el818^*	Fabian et al., 2020		
Genetic reagent (*Danio rerio*)	*Tg(Mmu.Sox10-Mmu.Fos:Cre)^zf384^*	Kague et al., 2012		
Genetic reagent (*Danio rerio*)	*Tg(actab2:loxP-BFP-STOP-loxP-dsRed)^sd27^*	Kobayashi et al., 2014		
Genetic reagent (*Danio rerio*)	*Tg(−6.5kdrl:mCherry)^ci5^*	Proulx et al., 2010		
Genetic reagent (*Danio rerio*)	*Tg(–5.0sox17:Cre-ERT2,myl7:DsRed)^sid1Tg^*	Hockman et al., 2017		
Genetic reagent (*Danio rerio*)	*Tg(cdh1:mlanYFP)^xt17Tg^*	Cronan and Tobin, 2019		
Genetic reagent (*Danio rerio*)	*gata3^b1075^*	Sheehan-Rooney et al., 2013		
Genetic reagent (*Danio rerio*)	*Tg(irx5a-p1:GFP, cryaa:Cerulean)^el859^*	This paper		See Materials and Methods, Section Zebrafish Lines
Recombinant DNA reagent	PCS2FA-transposase	Tol2Kit	PUBMED: 17937395 396.pCS2-transposase	
Recombinant DNA reagent	pDestTol2AB2-irx5a-p1-E1B:GFP_pA	This paper		See Materials and Methods, Section Zebrafish Lines
Sequence-based reagent	ucmaa RNAScope probe (*Danio rerio*); Channel 1	ACD Bio		
Sequence-based reagent	*gata2a* RNAScope probe (*Danio rerio*); Channel 1	ACD Bio		
Sequence-based reagent	*gata3* RNAScope probe (*Danio rerio*); Channel 2	ACD Bio		
Commercial assay or kit	In-Fusion HD Cloning Plus	Takara	Takara:638,910	
Commercial assay or kit	RNAScope Multiplex Fluorescent v2 Assay	ACD Bio	ACD Bio:323,100	
Other	Draq5 nuclear dye	Abcam	Abcam:Ab108410	See Materials and Methods, Section Imaging

### Zebrafish lines

The Institutional Animal Care and Use Committee of the University of Southern California approved all animal experiments (Protocol 20771). Zebrafish lines include *Tg(fli1a:eGFP)^y1^* (Lawson and Weinstein, 2002); *Tg(–4.9sox10:kikGR)^el2^* (Balczerski et al., 2012); *Tg(ucmaa_p1:GFP, cryaa:Cerulean)^el851^, Tg(gata3_p1:GFP, cryaa:Cerulean)^el858^*, and *Tg(fgf10b:nEOS)^el865^* (Fabian et al., 2022); *Tg(–5.0sox17:Cre-ERT2,myl7:DsRed)^sid1Tg^* (Hockman et al., 2017); *Tg*(*cdh1:mlanYFP)^xt17Tg^* (Cronan and Tobin, 2019); *Tg(–3.5ubb:loxP-STOP-loxP-mCherry)^el818^* (Fabian et al., 2020); *Tg(Mmu.Sox10-Mmu.Fos:Cre)^zf384^* (Kague et al., 2012); *Tg(actab2:loxP-BFP-STOP-loxP-dsRed)^sd27^* (Kobayashi et al., 2014); *Tg(−6.5kdrl:mCherry)^ci5^* (Proulx et al., 2010); and *gata3^b1075^* (Sheehan-Rooney et al., 2013). To generate *Tg(irx5a-p1:GFP, cryaa:Cerulean)^el859^*, we synthesized the intergenic peak associated with *irx5a* (chr7:35838071–35838577) using iDT gBlocks, cloned it into a modified pDestTol2AB2 construct containing the E1b minimal promoter, GFP, polyA, and the lens-specific *cryaa:Cerulean* marker using in-Fusion cloning (Takara Bio). We injected plasmid and Tol2 transposase RNA (5–10 ng/µL each) into one-cell stage zebrafish embryos and screened for founders at adulthood based on lens CFP expression in progeny. Two independent germline founders were identified that showed activity in gill pillar cells.

### Histology

Adult fish were fixed in 4% paraformaldehyde for 1 hr at 25°C followed by dissection of the gills and further fixation in 4% paraformaldehyde for 1 hr at 25°C. For pseudobranch dissection, adults were fixed in 4% paraformaldehyde at 4°C for 3 days prior to dissection. Alcian Blue staining was performed on whole tissue as previously described (Paul et al., 2016). Samples were imaged using a Leica DM2500 microscope. Image levels were adjusted in Adobe Illustrator.

### Photoconversion-based lineage tracing

To photoconvert mesenchyme in *sox10:kikGR; fli1a:GFP* fish at 1.5 dpf, we used the ROI function in ZEN software on a Zeiss LSM800 confocal microscope to expose dorsal first or second arch mesenchyme to UV light for 20 s. Imaging confirmed successful and specific photoconversion of kikGR from green to red fluorescence in the intended region. At 3.5 dpf, confocal imaging was used to assess contribution of photoconverted cells to pseudobranch mesenchyme. We included *fli1a:GFP* to help in identification of the pseudobranch bud. For *fgf10b:nEOS* photoconversion, we used the ROI function to expose nEOS-high expressing cells in the first or third pharyngeal pouches to UV light for 60 s, with immediate confocal imaging confirming intended photoconversion of nEOS from green to red fluorescence. At 5 dpf, confocal imaging was used to assess contribution of photoconverted cells to pseudobranch and gill epithelia. To confirm that nEOS-high expressing cells in the *fgf10b:nEOS* line were of endodermal original, we crossed these onto the *sox17:CreERT2; ubb:loxP-Stop-loxP-mCherry* transgenic background and treated embryos with 4-hydroxytamoxifen (Sigma) at 6.5 hpf to induce Cre recombination. We then imaged on the confocal microscope at 1.5 dpf to visualize co-localization of nEOS and mCherry. All results were independently confirmed in at least three animals.

### In situ hybridization

We performed in situ hybridization on whole embryos at 3 and 5 dpf and on paraffin sections from adult zebrafish heads using RNAscope probes synthesized by Advanced Cell Diagnostics in channel 1 (*ucmaa*, *gata2a*) and channel 2 (*gata3*). Samples were prepared by fixation in 4% paraformaldehyde overnight. Embryos were dehydrated in methanol and stored overnight before proceeding with the RNAScope Assay for Whole Zebrafish Embryos as described in the manufacturer’s protocols. Following fixation, the pseudobranch was dissected and mounted in 0.2% agarose in molds. Once solidified, agarose chips containing the pseudobranch were cut out of the mold, dehydrated, embedded in paraffin, and 5 µm sections were collected using a Shandon Finesse Me+ microtome (cat. no. 77500102). Paraformaldehyde-fixed paraffin-embedded sections were deparaffinized, and the RNAscope Fluorescent Multiplex V2 Assay was performed according to manufacturer’s protocols using an ACD HybEZ Hybridization oven. In situ patterns were confirmed in at least three independent animals, with exception of the *ucmaa* in situ that was performed on three separate sections of the same animal.

### Imaging

Images of whole-mount or section fluorescent in situ hybridizations and live transgenic fish were captured on a Zeiss LSM800 confocal microscope using ZEN software. For adult imaging of the *irx5a-p1:GFP* reporter, whole animals were euthanized and the pseudobranch and gills dissected out. The tissue was stained with Draq5 nuclear dye (Abcam) for 20 min to help identify pillar cells. Reported expression patterns for enhancer lines were confirmed in at least five animals.

## Data Availability

The current manuscript contains solely images, so no data have been generated for this manuscript. The n number for each image type is clearly stated in the manuscript. The following previously published dataset was used: CrumpG FabianP TsengK ChenH 2021Single-cell profiling of cranial neural crest diversification across a vertebrate lifetimeNCBI Gene Expression OmnibusGSE178969

## Additional files

MDAR checklist
